# Diagnostic Accuracy of Immature Platelet Fraction, Immature Reticulocyte Fraction, and Immature Granulocyte Percentage for Bacterial Sepsis: A Cross-Sectional Study

**DOI:** 10.7759/cureus.105416

**Published:** 2026-03-17

**Authors:** Arushi Choudhary, Shubhransu Patro, Sidharth S Pattnaik, Vibha Sharma, Parmarth Arora, Jyoti Prakash Sahoo

**Affiliations:** 1 Internal Medicine, Kalinga Institute of Medical Sciences, Bhubaneswar, IND; 2 Pharmacology, Kalinga Institute of Medical Sciences, Bhubaneswar, IND

**Keywords:** bacterial infection management, c-reactive protein (crp), diagnostic accuracy analysis, immature granulocyte percentage, immature platelet fraction, immature reticulocyte fractions, procalcitonin level, receiver operating characteristic curve (roc), sequential organ failure assessment (sofa), severe sepsis

## Abstract

Background and objectives: Sepsis-related mortality is a global health concern. It mandates early and accurate diagnosis of sepsis. Recently, the immature platelet fraction (IPF), immature reticulocyte fraction (IRF), and immature granulocyte percentage (IGP) have been evaluated as potential biomarkers for early sepsis detection. Hence, we conducted this study to evaluate IPF, IRF, and IGP of sepsis patients. We also evaluated the diagnostic accuracy and area under the curve (AUC) of these parameters.

Methods: This cross-sectional study was conducted from March 2023 to February 2025 at Kalinga Institute of Medical Sciences (KIMS), Bhubaneswar, India. We included adult patients with sepsis admitted to the KIMS medicine ICU during the study period. We recorded the following parameters assessed just after hospital admission: hemoglobin, white blood cell count (WBC), platelet count, IPF, IRF, IGP, procalcitonin, C-reactive protein (CRP), and Sequential Organ Failure Assessment (SOFA) score. All these parameters were reassessed on days 3 and 7. We then computed the sensitivity, specificity, and diagnostic accuracy of IPF, IRF, and IGP. R software (version 4.5.2, Vienna, Austria) was used for statistical analysis.

Results: We analyzed 1076 sepsis patients in this study. The study population had a median age of 58.0 (46.8-64.0) years. Of them, 608 (56.5%) were male participants. On day 0, the median values of IPF, IRF, and IGP of the study population were 4.98% (4.61-5.60), 2.79% (2.48-3.19), and 3.02% (2.40-3.95), respectively. The changes in these three parameters on day 7 were statistically significant (p < 0.001). The sensitivity values for IPF, IRF, and IGP were 0.933, 0.858, and 0.671, respectively. The specificity values for IPF, IRF, and IGP were 0.959, 0.919, and 0.708, respectively. The diagnostic accuracy values for IPF, IRF, and IGP were 0.954, 0.905, and 0.700, respectively. The AUC values for IPF, IRF, and IGP were 0.998 (0.997-0.999), 0.952 (0.934-0.971), and 0.708 (0.654-0.761), respectively.

Conclusion: The IPF, IRF, and IGP values at ICU admission reduced after seven days, indicating improvement in sepsis. Similarly, the SOFA scores were also reduced on day 7. The high values of IRF and IPF during admission implied increased erythropoietic and thrombopoietic actions in bone marrow. The surges in immature cell types indicated the initial stages of infection and the immunocompetent state of the patients. The high sensitivity, specificity, diagnostic accuracy, and AUC values of IPF, IRF, and IGP supported their potential as biomarkers for sepsis.

## Introduction

Sepsis is a life-threatening clinical syndrome triggered by a dysregulated host response to systemic infection, often leading to tissue damage, systemic inflammation, multiple organ failure, and death [1,2]. The burden of sepsis is higher in low- and middle-income countries (LMICs) like India than in high-income countries (HICs) [3]. Recent studies reported a 28-36% mortality rate due to sepsis in India [4,5]. Sepsis mortality ranges from 15% to 25% in developed countries [6]. Early diagnosis of sepsis is crucial to lessen the burden on global health.

The Sepsis-3 criteria [7] define sepsis as a life-threatening organ dysfunction caused by a dysregulated host response to infection, quantified using the Sequential Organ Failure Assessment (SOFA) score [8]. Blood culture reports take 48-72 hours to confirm the source of infection [9]. False negative reports delay sepsis management and prolong hospital stays [9,10]. This diagnostic ambiguity necessitates the development of cost-effective and rapid biomarkers in resource-constrained settings.

Researchers have explored a number of biomarkers, such as procalcitonin (PCT) and C-reactive protein (CRP), to aid in the early detection and treatment of sepsis [11,12]. PCT is useful for differentiating between bacterial and viral infections and systemic inflammatory reactions [11,13]. But in non-infectious inflammatory situations, such as burns, trauma, or major surgeries, PCT levels might be erroneously raised, which lowers its specificity in critically ill patients [11]. CRP is a non-specific acute-phase reactant. Although easily accessible, CRP levels are elevated in infectious, inflammatory, and autoimmune diseases [11,14]. Serum lactate is a common marker of tissue hypoxia but is not infection-specific [15]. Furthermore, the extensive therapeutic usage of PCT and interleukin-6 (IL-6) is limited in underdeveloped nations due to their high cost and limited availability [11,16]. Due to these restrictions, hematological indices like immature platelet fraction (IPF), immature reticulocyte fraction (IRF), and immature granulocyte percentage (IGP) are being explored as potential biomarkers for early detection of bacterial sepsis [17-19]. These markers are appropriate for low-resource settings since they are regularly produced by automated hematology analyzers [17,18].

The percentage of freshly released, immature platelets in the peripheral circulation is known as IPF [17,19]. These platelets are larger, RNA-rich, and metabolically active, and their increase reflects bone marrow response to thrombocytopenia, systemic inflammation, or infection [20]. Systemic inflammation in sepsis increases platelet consumption and destruction, which, in turn, prompts the bone marrow to release immature platelets into the blood [17,19,20]. Park et al. demonstrated that IPF had a high diagnostic sensitivity in distinguishing septic from non-septic patients [21]. The normal range of IPF spans between 1% and 7% [22]. IRF measures the proportion of immature reticulocytes among total reticulocytes [17,19]. Reticulocytes are the young red blood cells that retain residual RNA. IRF is a sensitive indicator of bone marrow erythropoietic activity. In the context of sepsis, IRF may reflect the host's bone marrow response to systemic inflammation and oxidative stress [17,23]. IRF values are higher in sepsis patients than in those without sepsis [17]. Immature granulocytes include promyelocytes, myelocytes, and metamyelocytes, which are typically restricted to the bone marrow but may appear in peripheral circulation in response to infection or inflammation [18]. IGP quantifies the percentage of these immature cells, offering an early marker of infection [18,24]. IGP > 3% is considered a biomarker for sepsis [25].

We designed this study to evaluate the IPF, IRF, and IGP values of sepsis patients on days 0, 3, and 7. We additionally assessed the diagnostic accuracy of these three parameters.

## Materials and methods

We conducted this cross-sectional study from March 2023 to February 2025 at the Kalinga Institute of Medical Science (KIMS), Bhubaneswar, India. We received ethical approval from the Institutional Ethics Committee (KIIT/KIMS/IEC/1166/2023, dated February 27, 2023) before beginning the study.

Study criteria

We included adult patients of both sexes admitted to our medicine intensive care unit (ICU) during the study period, fulfilling the Sepsis-3 criteria [7]. We excluded patients with any malignancy, autoimmune disease, immunocompromised state, ongoing steroids, antibiotics, anticancer drugs, or blood component transfusion. The patients who were diagnosed with sepsis before ICU admission and those on antibiotics within the last seven days were excluded. We also excluded the patients referred from other hospitals and those who died within or stayed for less than seven days.

Study procedure

We recorded the participant data from their case sheets during their discharge from the hospital. The sociodemographic details, i.e., age, gender, socioeconomic status, and marital status of all participants, were recorded. The Kuppuswamy classification was used to categorize participants' socioeconomic status [26]. We noted the following parameters assessed immediately after hospital admission: hemoglobin, white blood cell count (WBC), platelet count, IPF, IRF, IGP, PCT, CRP, and SOFA score. All these parameters were reassessed on days 3 and 7. The trends of all parameters across all time points were analyzed. We evaluated the sensitivity, specificity, and diagnostic accuracy of IPF, IRF, and IGP.

Statistical analysis

We employed non-probability consecutive sampling for this cross-sectional study. The normality of the data distribution was checked using the Shapiro-Wilk test. Categorical data were presented as frequencies and proportions. Continuous data were reported as median and interquartile range (IQR). Continuous and categorical data were assessed using the Kruskal-Wallis test and the chi-square test, respectively. For the post-hoc assessment, we used the Bonferroni correction. Receiver operating characteristic (ROC) analysis was performed for IPF, IRF, and IGP. The area under the curve (AUC) was evaluated with 95% confidence intervals (CIs) for IPF, IRF, and IGP. Version 4.5.2 of the R software (Vienna, Austria) was used for data computation [27]. Statistical significance was set at p ≤ 0.05.

## Results

During the study period, 2198 patients were admitted to the medicine ICU with sepsis. Four hundred twenty-seven (19.4%) patients had sepsis before their ICU admission. Three hundred ninety-two (17.8%) patients had sepsis before their ICU admission. Three hundred three (13.8%) subjects had incomplete details in their case sheets. The remaining 1076 (49.0%) individuals were analyzed in this study. Table 1 presents the sociodemographic characteristics of the 1076 participants. The study population had a median age of 58.0 (46.8-64.0) years. Of them, 608 (56.5%) were male participants.

**Table 1:** Demographic details of the study participants (n = 1076) Categorical data were presented as frequencies and proportions. Continuous data were presented as median and IQR. IQR: interquartile range.

Parameters	Value
Age (years)	58.0 (46.8-64.0)
Elderly (age > 60 years)	241 (22.4%)
Male	608 (56.5%)
Marital status	
Married	986 (91.6%)
Unmarried	33 (3.1%)
Divorced/widowed	57 (5.3%)
Socioeconomic status	
Low	549 (51.0%)
Lower middle	484 (45.0%)
Upper middle	43 (4.0%)

**Figure 1:**
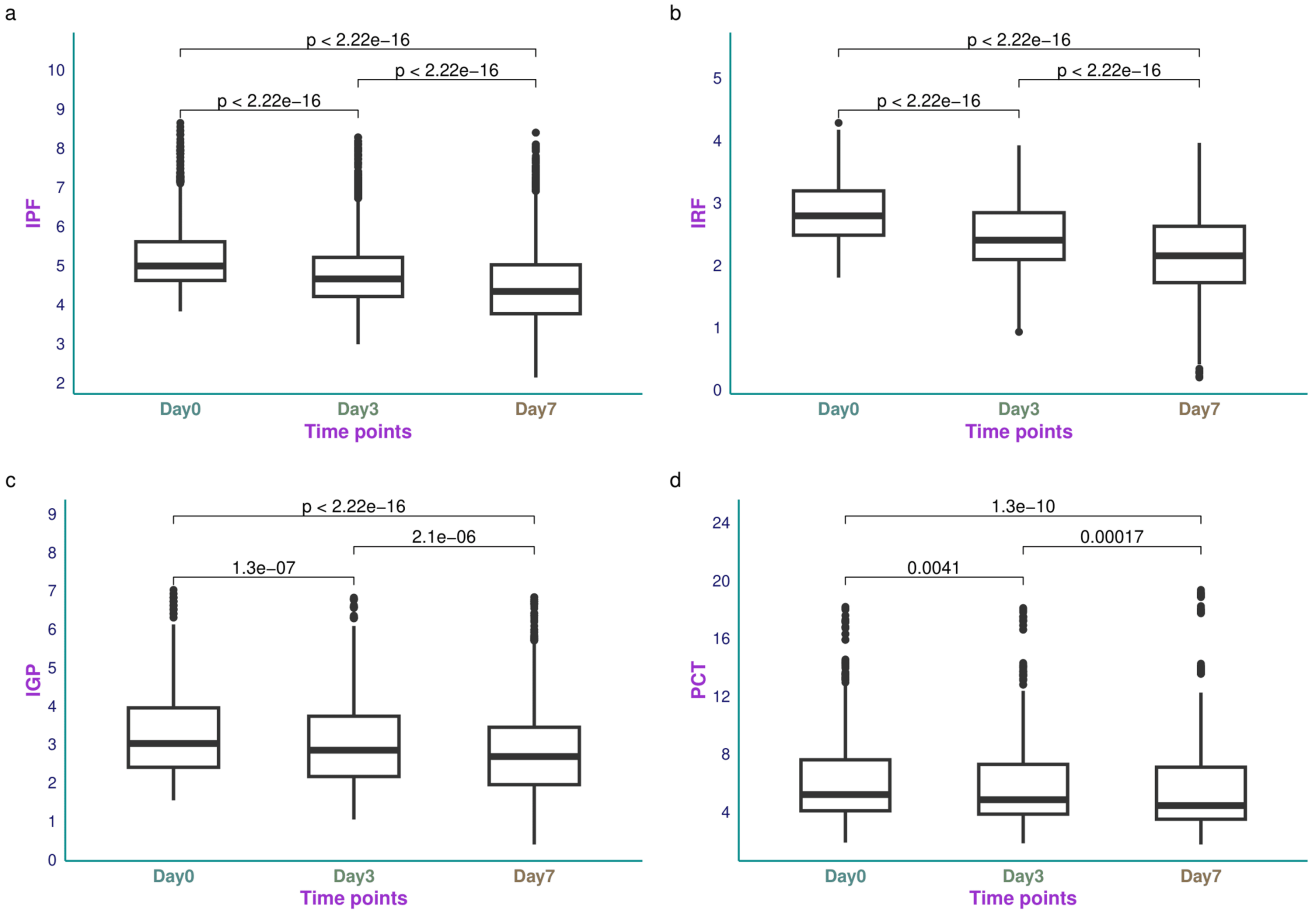
IPF, IRF, IGP, and PCT values of the study participants The box-and-whisker plots present the IPF, IRF, IGP, and PCT values at different time points in panels a-d, respectively. IPF, IRF, and IGP were shown as percentages. PCT values were reported in ng/mL. The analyses were done with the Kruskal-Wallis test. The post-hoc assessment was conducted with the Bonferroni correction. The statistical significance was set at p < 0.05. IPF: immature platelet fraction; IRF: immature reticulocyte fraction; IGP: immature granulocyte percentage; PCT: procalcitonin.

Figure 1 illustrates the IPF, IRF, IGP, and PCT values of the study population. The median IPF of the study population was 4.98% (4.61-5.60%) on day 0 (Figure 1a). The reduction in IPF values over seven days was statistically significant (p < 0.001). The median IRF of the study population was 2.79% (2.48-3.19%) on day 0 (Figure 1b). The reduction in IRF values over seven days was also statistically significant (p < 0.001). The median IGP of the study population was 3.02% (2.40-3.95%) on day 0 (Figure 1c). The reduction in IGP values over seven days was statistically significant (p < 0.001). The median PCT value of the study population was 5.20 (4.08-7.60) ng/mL (Figure 1d). The change in PCT values over seven days was also statistically significant (p < 0.001). Table 2 shows the IPF, IRF, IGP, and PCT values of the study population on days 0, 3, and 7.

**Table 2:** Blood parameters and SOFA scores of the study participants Continuous data were expressed as median and IQR. The analyses were done with the Kruskal-Wallis test, and the H-values for the same were provided. The statistical significance was set at p < 0.05. IPF: immature platelet fraction; IRF: immature reticulocyte fraction; IGP: immature granulocyte percentage; PCT: procalcitonin; WBC: white blood cell count; CRP: C-reactive protein; SOFA: Sequential Organ Failure Assessment; IQR: interquartile range.

Parameters	Day 0	Day 3	Day 7	H-value	p-value
IPF (%)	4.98 (4.61-5.60)	4.65 (4.40-5.20)	4.33 (3.76-5.01)	362.98	< 0.001
IRF (%)	2.79 (2.48-3.19)	2.40 (2.09-2.84)	2.15 (1.72-2.62)	600.55	< 0.001
IGP (%)	3.02 (2.40-3.95)	2.84 (2.16-3.73)	2.68 (1.95-3.44)	96.05	< 0.001
PCT (ng/mL)	5.20 (4.08-7.60)	4.85 (3.85-7.29)	4.44 (3.50-7.09)	42.40	< 0.001
Hemoglobin (g/dL)	10.25 (9.00-11.03)	10.20 (8.90-11.10)	10.10 (9.00-11.00)	0.82	0.66
WBC (× 10^9^/L)	17.68 (15.40-20.44)	16.50 (14.77-18.80)	15.20 (13.20-17.31)	246.01	< 0.001
Platelet count (× 10^9^/L)	258.5 (215.0-319.0)	251.0 (207.0-312.0)	242.0 (201.0-308.0)	21.15	< 0.001
CRP (mg/L)	52.20 (32.70-71.45)	47.85 (31.18-64.00)	42.30 (27.78-58.60)	74.76	< 0.001
SOFA score	4.0 (3.0-6.0)	5.0 (2.0-7.0)	4.0 (2.0-6.0)	24.25	< 0.001

**Figure 2:**
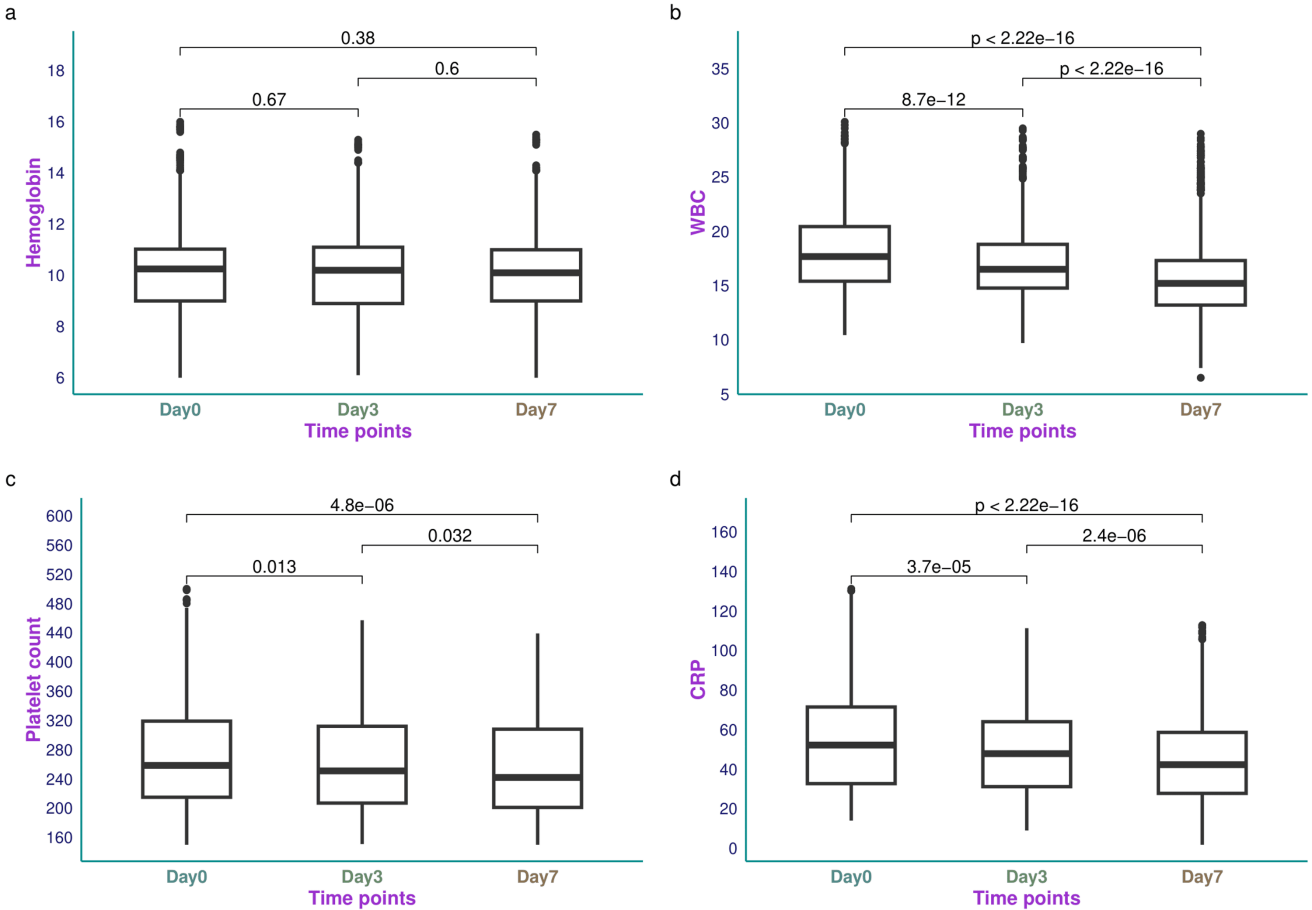
Hemoglobin, WBC, platelet count, and CRP values of the study participants The box-and-whisker plots present the hemoglobin, WBC, platelet count, and CRP values at different time points in panels a-d, respectively. Hemoglobin values were expressed in g/dL. WBC and platelet counts were expressed as 10^9^/L. The CRP values were expressed as mg/L. The analyses were done with the Kruskal-Wallis test. The post-hoc assessment was conducted with the Bonferroni correction. The statistical significance was set at p < 0.05. WBC: white blood cell count; CRP: C-reactive protein.

Figure 2 illustrates the hemoglobin, WBC, platelet count, and CRP values of the study population. The median hemoglobin value of the study population on day 0 was 10.25 (9.00-11.03) g/dL (Figure 2a). The change in hemoglobin values after seven days was not statistically significant (p = 0.66). The median WBC count for study participants on day 0 was 17.68 (15.40-20.44) × 10^9^/L (Figure 2b). The change in WBC after seven days was statistically significant (p < 0.001). The median platelet count of the study participants on day 0 was 258.5 (215.0-319.0) × 10^9^/L (Figure 2c). The change in platelet count after seven days was statistically significant (p < 0.001). The median CRP value of the study population on day 0 was 52.20 (32.70-71.45) mg/L (Figure 2d). The change in platelet count after seven days was statistically significant (p < 0.001). Table 2 shows the hemoglobin, WBC, platelet count, and CRP values of the study population on days 0, 3, and 7.

**Figure 3:**
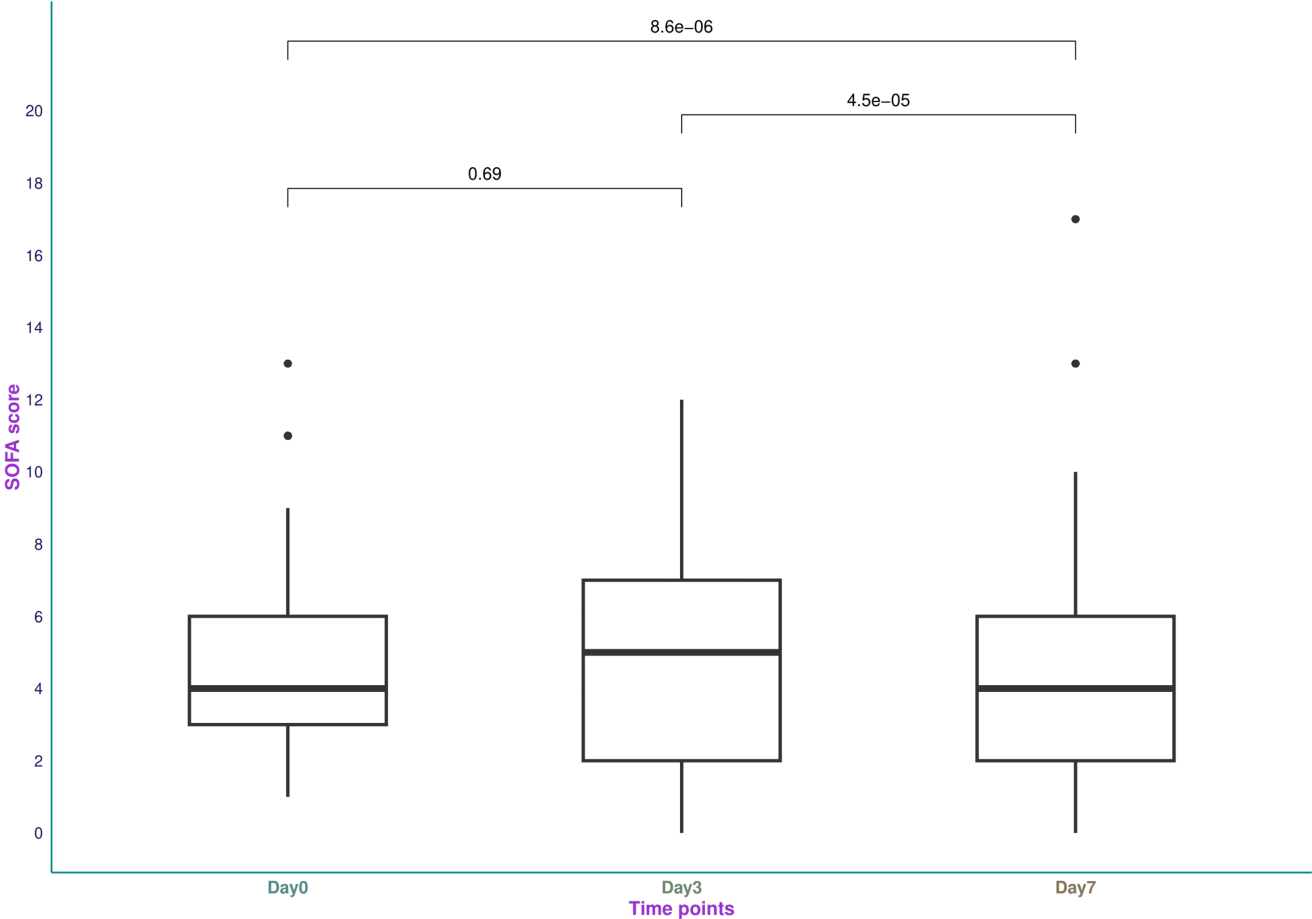
SOFA scores of the study participants The box-and-whisker plots present the SOFA scores at different time points. The analyses were done with the Kruskal-Wallis test. The post-hoc assessment was conducted with the Bonferroni correction. The statistical significance was set at p < 0.05. SOFA: Sequential Organ Failure Assessment.

Figure 3 and Table 2 illustrate the SOFA scores of the study population. The median SOFA scores on days 0, 3, and 7 were 4.0 (3.0-6.0), 5.0 (2.0-7.0), and 4.0 (2.0-6.0), respectively. The change in SOFA scores after seven days was statistically significant (p < 0.001).

**Figure 4:**
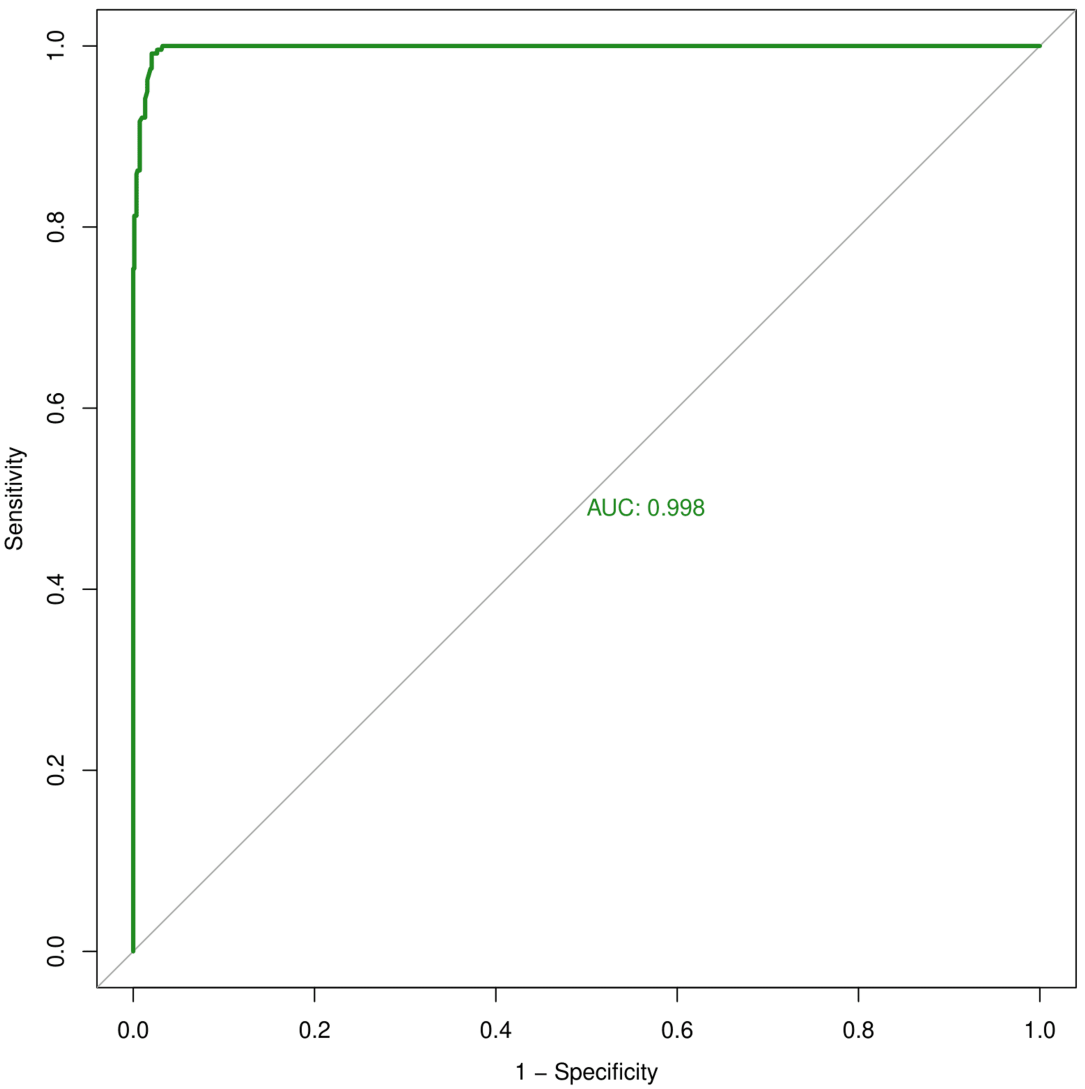
ROC curve for IPF The X- and Y-axes represent 1 - specificity and sensitivity values of IPF, respectively. The AUC was 0.998. IPF: immature platelet fraction; ROC: receiver operating characteristic curve; AUC: area under the curve.

**Figure 5:**
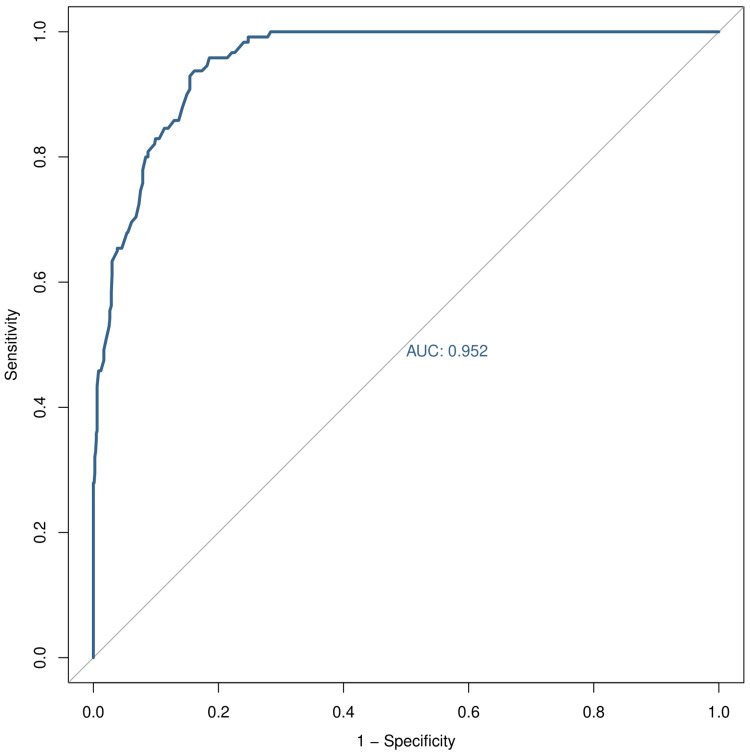
ROC curve for IRF The X- and Y-axes represent 1 - specificity and sensitivity values of IRF, respectively. The AUC was 0.952. IRF: immature reticulocyte fraction; ROC: receiver operating characteristic curve; AUC: area under the curve.

**Figure 6:**
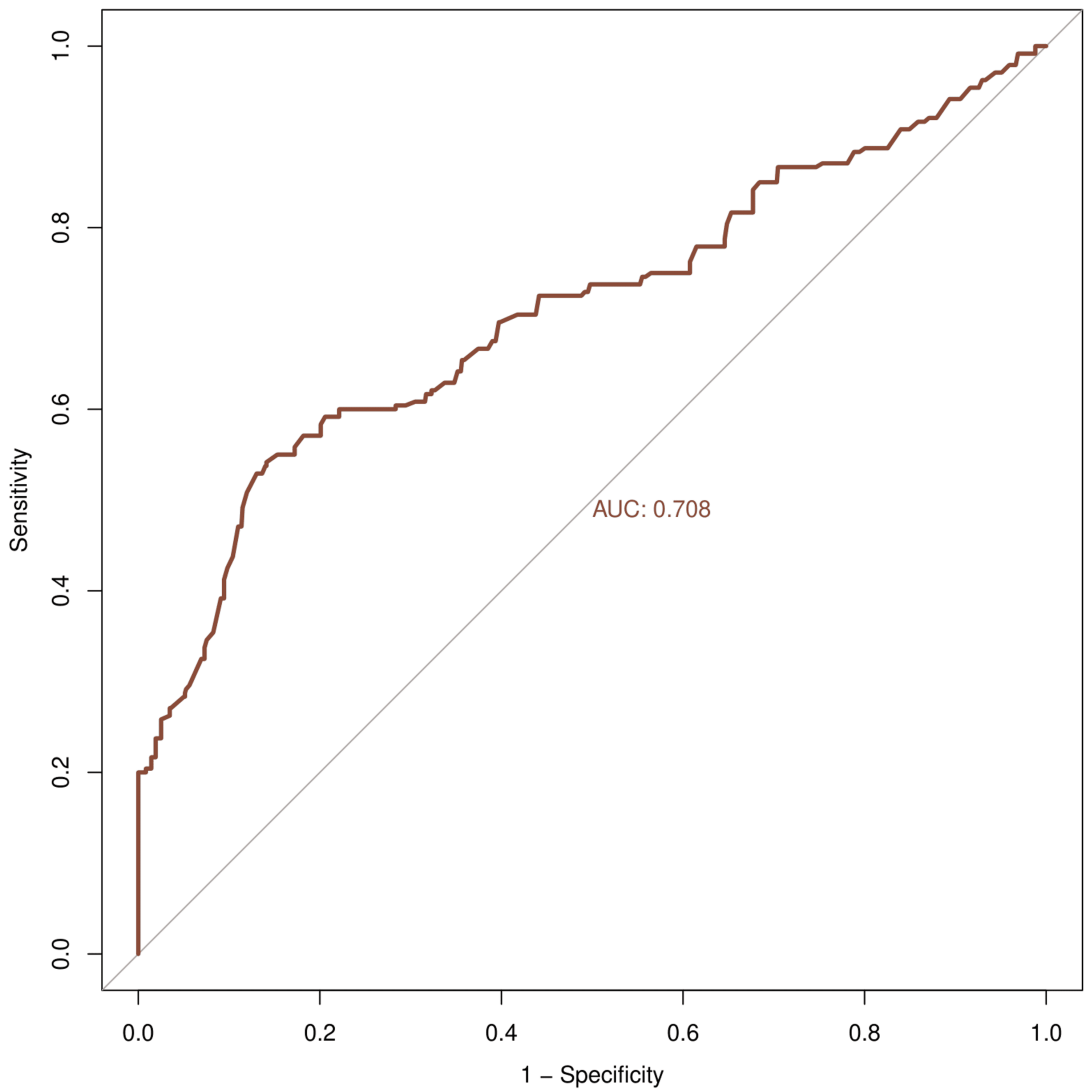
ROC curve for IGP The X- and Y-axes represent 1 - specificity and sensitivity values of IGP, respectively. The AUC was 0.708. IGP: immature granulocyte percentage; ROC: receiver operating characteristic curve; AUC: area under the curve.

Figures 4-6 illustrate the ROC curve of IPF, IRF, and IGP, respectively. Table 3 shows the sensitivity, specificity, diagnostic accuracy, AUC, and its 95% CI for IPF, IRF, and IGP. The sensitivity values for IPF, IRF, and IGP were 0.933, 0.858, and 0.671, respectively. The specificity values for IPF, IRF, and IGP were 0.959, 0.919, and 0.708, respectively. The diagnostic accuracy values for IPF, IRF, and IGP were 0.954, 0.905, and 0.700, respectively. The AUC values for IPF, IRF, and IGP were 0.998 (0.997-0.999), 0.952 (0.934-0.971), and 0.708 (0.654-0.761), respectively.

**Table 3:** Diagnostic accuracy and AUC of IPF, IRF, and IGP The ranges for sensitivity, specificity, diagnostic accuracy, and AUC for IPF, IRF, and IGP were 0-1. IPF: immature platelet fraction; IRF: immature reticulocyte fraction; IGP: immature granulocyte percentage; AUC: area under the curve; CI: confidence interval.

Parameters	Sensitivity	Specificity	Diagnostic accuracy	AUC	95% CI for AUC
IPF	0.933	0.959	0.954	0.998	0.997-0.999
IRF	0.858	0.919	0.905	0.952	0.934-0.971
IGP	0.671	0.708	0.700	0.708	0.654-0.761

## Discussion

In this cross-sectional study, we analyzed the levels of IPF, IRF, and IGP. We also calculated their diagnostic accuracy values. These three parameters have emerged as cost-effective, easily accessible, and novel biomarkers for sepsis [17-19]. Sepsis remains a major global health concern, particularly in the ICU setting, where rapid progression to organ dysfunction or septic shock can occur if not promptly recognized [2,10,28]. The key to improving sepsis outcomes lies in early and accurate diagnosis [10,19,28]. Traditional diagnostic markers such as WBC, lactate, and CRP often lack the sensitivity and specificity required for timely sepsis detection [11,12,14].

IPF illustrates the rate of thrombopoiesis. Elevated IPF levels are associated with increased platelet turnover, which is frequently observed in the early phases of sepsis due to consumption coagulopathy and bone marrow response [29]. Hubert et al. found that IPF levels were significantly elevated in patients with sepsis compared to those without sepsis, suggesting its strong diagnostic value [19]. IRF represents the proportion of immature reticulocytes released from the bone marrow due to stress erythropoiesis. While IRF is traditionally used in hematological disorders, recent research indicates its elevation in critically ill patients may reflect systemic stress or hypoxia seen in sepsis [30]. Recent studies have shown that elevated IGP levels have greater sensitivity than WBC in predicting sepsis progression [18,31].

The significant elevation of IGP, IPF, and IRF in septic patients reflects the heightened demand for granulocyte, platelet, and erythroid precursors in response to systemic infection [17,25,29,31]. These changes represent a reactive marrow response to the inflammatory milieu and cytokine storm that typify sepsis [18,28,30].

The use of IGP and IPF, obtainable from routine complete blood count differentials using modern hematology analyzers, offers an attractive alternative. These markers can be reported within minutes, without additional sampling or cost, making them ideal for time-sensitive settings like the emergency department or ICU triage. Their early rise reflects a real-time marrow response that precedes systemic organ dysfunction, potentially enabling preclinical detection of sepsis and earlier escalation of care. Additionally, early risk stratification using IGP and IPF could help reduce inappropriate antibiotic use by more accurately identifying bacterial infections, thus supporting antimicrobial stewardship, another key goal in modern critical care.

Strengths and limitations

The major strengths of our study were the analyses of IPF, IRF, and IGP among sepsis patients. We also evaluated their diagnostic accuracy values. Our study has certain limitations as well. First, this was a single-center study. Second, prognostic outcomes, such as ICU length of stay, organ dysfunction, and patient outcomes, were beyond the scope of this study. Third, the effects of comorbidities and concurrent drugs on the clinical characteristics of sepsis patients were not evaluated. Fourth, we did not include serum lactate and IL-6 levels due to laboratory constraints.

## Conclusions

The high values of IPF, IRF, and IGP during ICU admission decreased by day 7. The SOFA scores were also decreased on day 7. The reductions in these parameters indicated improvement in sepsis over time. The initial high values of IPF and IRF indicated increased thrombopoietic and erythropoietic activity in bone marrow in response to infection, inflammation, and sepsis, respectively. The rises in immature cell variants reflected the early stages of infection and the immunocompetent state of the patients. The high sensitivity, specificity, diagnostic accuracy, and AUC values of IPF, IRF, and IGP supported their potential as biomarkers for sepsis. We recommend prospective studies with larger sample sizes to compare and contrast these parameters in predicting sepsis progression and its prognosis.
